# Cardiac risk in recovered Covid-19 patients evaluated by ^123^I-mIBG

**DOI:** 10.1038/s41598-025-02212-7

**Published:** 2025-05-23

**Authors:** Alessandro Liebich, Gabriel Sheikh, Ralph. A. Bundschuh, Malte Kircher, Alexander Dierks, Bernd Nittbaur, Philip Raake, Maximilian Rieger, Takahiro Higuchi, Christian H. Pfob, Constantin Lapa

**Affiliations:** 1 Nuclear Medicine, Faculty of Medicine, University of Augsburg, Augsburg, Germany; 2 Department of Nuclear Medicine, LMU Munich, Munich, Germany; 3 Faculty of Medicine, University of Augsburg, Augsburg, Germany; 4 Comprehensive Heart Failure Center (CHFC) and Department of Nuclear Medicine, University Hospital Wuerzburg, Wuerzburg, Germany; 5 Klinik für Nuklearmedizin, Universitätsklinikum Augsburg, Stenglinstraße 2, 86156 Augsburg, Bavaria Germany

**Keywords:** Cardiology, Medical research

## Abstract

To determine whether cardiac sympathetic nervous dysfunction is present, in this single center prospective, non-randomized trial non-invasive SPECT/CT imaging using the radiotracer ^123^I-metaiodobenzylguanidine was performed in 33 recovered COVID-19 patients without pre-existing cardiac conditions. Increased cardiac sympathetic activity, as indicated by late HMR, was observed in 67.7% of patients. At 6–8 months, 82% of these subjects (27/33) received follow-up, and cardiac sympathetic innervation abnormalities were still present in 70.4% (19/27). Additionally, at 12–15 months post-diagnosis, persistently abnormal HMRs were found in 9 individuals who initially had abnormal sympathetic innervation. Further follow-up is needed to investigate potential long-term cardiovascular consequences of COVID-19.

## Introduction

COVID-19 patients without underlying heart disease can develop heart failure, myocarditis or cardiac arrhythmias^1,2^. Significant cardiac involvement seems to occur independently from the severity of the initial disease pattern and may persist during the long-term recovery period^3^. Cardiac autonomic dysregulation is central to the progression of most cardiovascular diseases, including hypertension, heart failure, arrhythmias and myocardial infarction and enhanced sympathetic activity is a negative prognostic factor for both morbidity and mortality associated with arrhythmias and sudden death^4–6^. Enhanced cardiac sympathetic activity, measured non-invasively with the radiotracer ^123^I-metaiodobenzylguanidine (^123^I-mIBG, AdreView™, GE Healthcare, Arlington Heights, IL, USA), a norepinephrine analogue, is a well-established negative prognostic factor in heart failure, and semi-quantitative parameters such as the heart-to-mediastinum ratio (HMR) are used clinically for the prediction of disease progression, risk for ventricular arrhythmias and sudden cardiac death^7,8^. One study found an annualized mortality rate of < 1% in heart failure patients with New York Heart Association (NYHA) functional class I-II and HMR ≥ 2.0, irrespective of left ventricular ejection fraction (LVEF) and age, while the mortality rate for patients with NYHA class III–IV was 4 to 6 times higher with HMR < 1.4, independent of LVEF^9^. The ADMIRE-HF (AdreView Myocardial Imaging for Risk Evaluation in Heart Failure) trial could demonstrate that patients with heart failure and a HMR ≥ 1.6 had a significantly lower risk of disease progression, arrhythmic events and cardiac death compared to patients with a HMR < 1.6.^10^. Given the large number of COVID-19 survivors, enhanced surveillance and treatment for those with significant cardiac abnormalities could probably substantially lower the burden of subsequent morbidity and mortality. Since several investigations have shown that impaired cardiac mIBG activity can improve in response to current medical management using beta-adrenoceptor blockers and renin–angiotensin–aldosterone system inhibitors^11,12^, a quantitative assessment of this system should have an obvious value as an additional piece of relevant medical information. To evaluate the presence of cardiac sympathetic nervous dysfunction in recovered sub-acute COVID-19, non-invasive imaging using mIBG scintigraphy was performed in COVID-19 patients without pre-existing cardiac conditions.

## Materials and methods

In this prospective, non-randomized, single-arm trial 33 recovered sub-acute COVID-19 patients (14 men and 19 women; median age, 40 years; range, 21–66 years) without any known cardiac, renal, neurological or metabolic disease were included from 2021 to 2023. Especially heart failure, coronary heart disease, diabetes, hypertension, stroke or kidney failure were not present in any patient. None of the individuals were taking drugs interacting with mIBG uptake as detailed in current guidelines^13^. None of them had been hospitalized to intensive care unit due to COVID-19. All subjects gave written informed consent for inclusion before participating in the study. The study was conducted in accordance with the Declaration of Helsinki. The protocol was approved by the Ethics Committee of Ludwig-Maximilians-University Munich (Project identification code: 20-1140) and announced in the German Clinical Trials Register (Registration number: DRKS00023773).

All subjects were initially tested positive for COVID-19 using real-time-polymerase chain reaction (Hologic Aptima™ SARS-CoV-2 assay). All patients underwent ^123^I-mIBG- SPECT/CT, echocardiography as well as serum measurement of cardiac enzymes, renal retention parameters and electrolytes 0 to 3 months after recovery (^123^I-mIBG was performed 49 ± 21 days after recovery). In 82% of these patients (27 out of 33), follow-up was performed 6 to 8 months after diagnosis. 6 patients did not receive follow-up mostly due to time and scheduling conflicts. In addition, 9 patients with persistent increased cardiac sympathetic activity underwent an additional ^123^I-mIBG-scan 12 to 15 months after recovery. No therapy was initiated in any patient after their initial consultation.

SPECT/CT scans were acquired 15 min and 4 h post-injection of 370 MBq ^123^I-mIBG using a dedicated SPECT/CT system (GE OptimaTM NM/CT670, GE Healthcare, Milwaukee, WI, USA) or Siemens Symbia T2, Siemens Healthineers, Erlangen, Germany). A low-energy-high-resolution (LEHR) collimator was used in all studies. For quantification of results, a circular region of interest (ROI) was placed over the heart and mediastinum to calculate late (4-h) HMR. It is known that the correct cut-off for cardiac ^123^I-mIBG image interpretation is affected by population differences and technical factors^14^. Late HMR is known to correlate consistently with survival over a wide range of HMRs from 1.1 to 2.1. In heart-failure, patients with a late HMR > 2.10 can be described as low risk according to the overall survival rate, therefore we choose 2.1 as cut-off^15^. Echocardiographic global longitudinal strain analysis (GLS) analysis was available for 82% of the patients (27 out of 33) after recovery and for 85% of the patients (23 out of 27) at 6–8 months follow up.

## Results

**Fig. 1:**
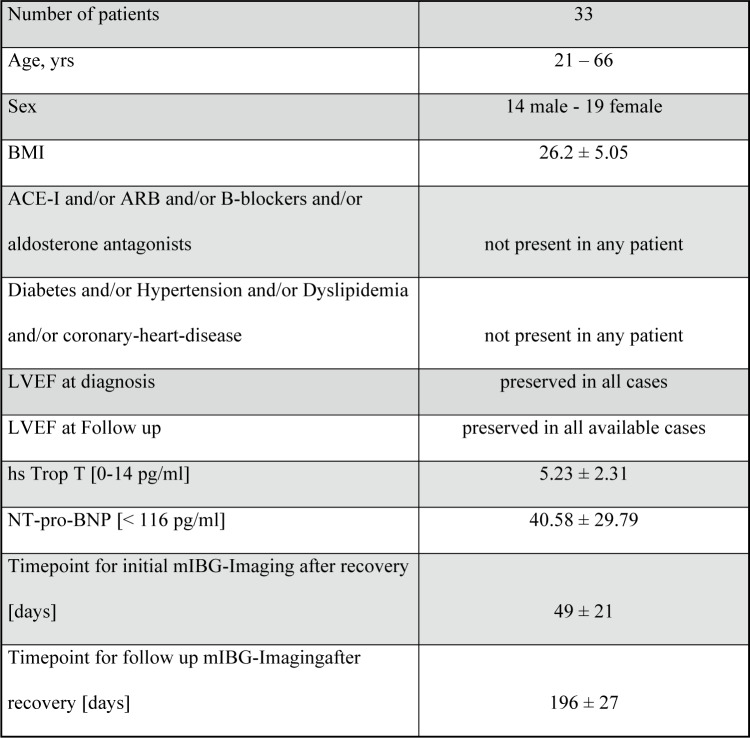
Patient characteristics.

Previous cardiac, renal, neurological or metabolic disease was not present in any patient and the mean body mass index (BMI) was 26.2 (± 5.1), indicating a population with a low a priori cardiovascular risk. LVEF and cardiac enzyme levels were normal in all cases, both at baseline and at follow-up. Echocardiagraphic strain analysis remained normal after recovery (–22.5 ± 1.7) and at 6–8 months follow up (− 22.3 ± 2.0). Figure 1 provides an overview on the patients´ characteristics.

Reduced cardiac mIBG uptake as indicated by late HMR (mean HMR, 1.8 ± 0.2), was observed in 67.7% of the patients (23 out of 33; 95% CI 0.54–0.854). At 6–8 months follow-up, reduced cardiac mIBG uptake was still present in 70.4% (19 out of 27; 95% CI 0.532–0.876) of patients, with a mean HMR of 1.74 ± 0.27. Only 3 patients with initially reduced cardiac mIBG uptake showed normalization at 6–8 months follow up, 4 patients with initially normal cardiac mIBG uptake showed reduced uptake at 6–8 months follow-up, and 15 subjects demonstrated persistently abnormal HMRs (mean HMR, 1.76 ± 0.25). Additionally, 9 patients with initially abnormal sympathetic innervation underwent follow-up 12–15 months post-diagnosis, and all were found to have persistently abnormal HMRs (mean HMR 1.69 ± 0.3).

**Fig. 2:**
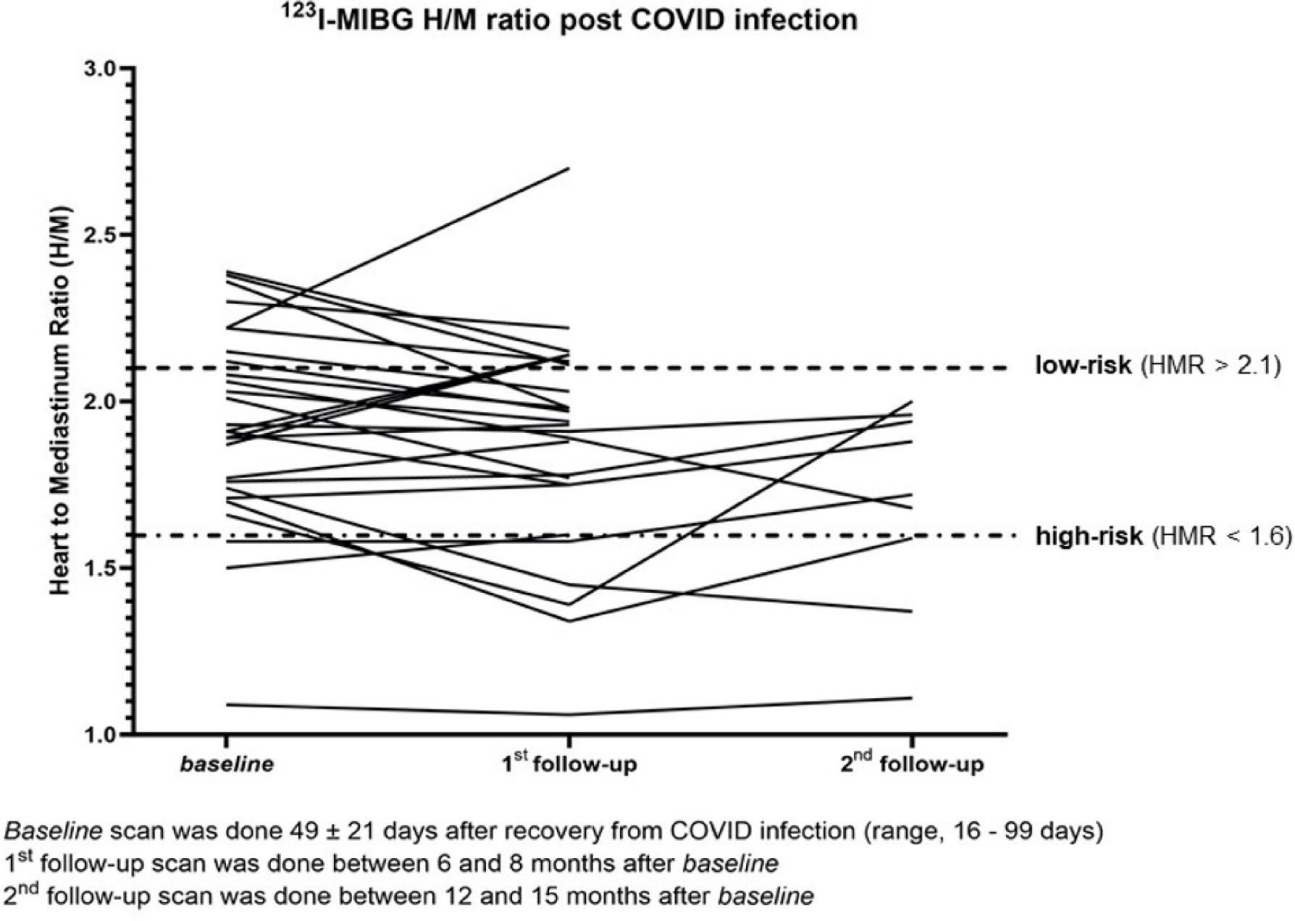
Late Heart-to-mediastinum ratio over time for every patient after recovery (baseline), 6–8 month follow-up (1st follow-up) and 12–15 month follow-up (2nd follow-up).

Noteworthy, choosing a late HMR ≤ 1.6 as a potential marker for patients at high risk for cardiac events (as defined by the ADMIRE-HF trial) pathologically reduced cardiac mIBG uptake was observed in 9.1% (3 out of 33) of subjects at the initial imaging and increased to 18.5% (5 out of 27) of patients at 6–8 months. At 12–15 months post-diagnosis, 3 of those individuals showed persistently abnormal HMRs. Individual HMRs for each subject and imaging time point are shown in Fig. 2.

**Fig. 3:**
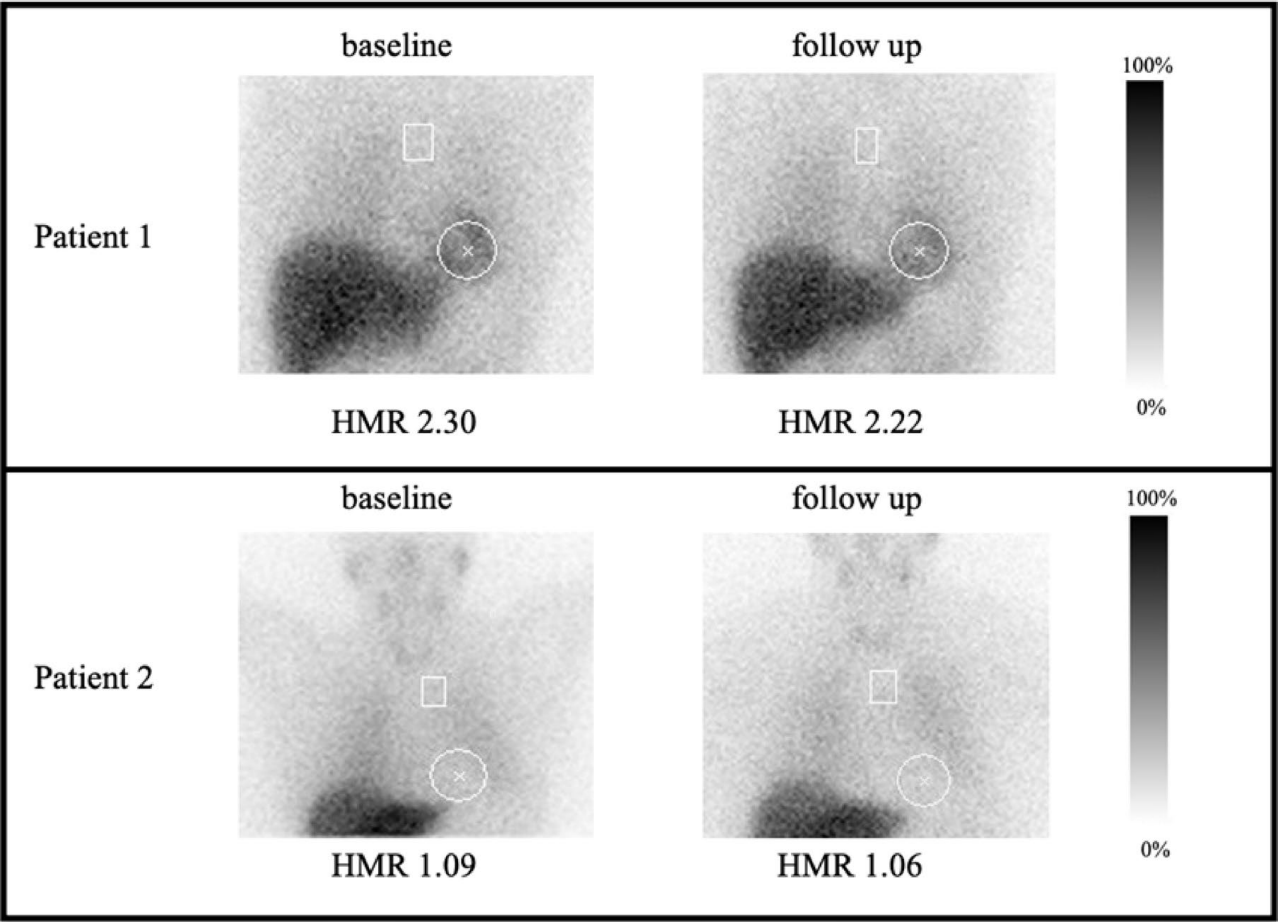
Planar scintigraphy images 4 h post-injection of 370 MBq ^123^I-mIBG of two examined patients. Patient 1 demonstrates a normal late HMR, both initially after recovery (upper left) as well as at 6–8 month follow-up (upper right) with preserved cardiac mIBG uptake. In contrast, Patient 2 shows decreased cardiac mIBG uptake, resulting in a decreased late HMR at diagnosis (bottom left) with persistence at follow-up (bottom right).

SPECT/CT showed homogenously decreased uptake in all cases with reduced cardiac mIBG uptake, without local inhomogeneity (Fig. 3).

There was no significant correlation between LVEF and HMR as LVEF was normal in all cases. Furthermore, there was no significant correlation between NT-pro-BNP (*p* = 0.95), troponin levels (*p* = 0.86), GLS (*p* = 0.89), age (*p* = 0.81), BMI (*p* = 0.88) or sex (*p* = 0.91) and HMR. During follow-up, no clinical deterioration in terms of arrhythmias, heart failure or sudden death was observed.

Regarding the prevalence of different coronavirus subtypes during the pandemic, no differences in HMR as potential hints to specific cardiac effects of the various viral variants were detected over the period of time.

## Discussion

This is, to our knowledge, the first report of increased cardiac sympathetic activity in COVID-19 with persistent increased cardiac sympathetic activity observed in two thirds of enrolled patients. None of the subjects had a history of prior cardiac disease, and relevant factors confounding sympathetic cardiac activity such as diabetes mellitus, arterial hypertension or interfering drugs were excluded.

At 6 (-8) months follow-up, reduced cardiac mIBG uptake was still present in 19 out of 27 patients, only 3 patients with initially reduced cardiac mIBG uptake showed normalization at 6–8 months follow up. In addition all nine subjects with initially reduced late HMR who underwent another imaging at 12–15 months demonstrated persistent pathological values. Choosing -in analogy to the ADMIRE-HF trial- a late HMR ≤ 1.6 as a potential marker for patients at high risk for cardiac events, pathologically reduced cardiac mIBG uptake was initially observed in roughly 10% of subjects and increased to even 18.5% (5 out of 27) of patients at 6–8 months. At 12–15 months post-diagnosis, 3 of those continued to show persistently abnormal HMRs. These findings indicate that enhanced cardiac sympathetic activity tended to persist over time. In addition, as only 3 patients showed normalization at 6–8 months follow up, we believe that the observed changes to cardiac sympathetic activity might be long-term (or even permanent) in a high proportion of patients, similar to other (long-)COVID-related conditions However, further long-term follow-up to assess the clinical significance of abnormal HMR is mandatory.

In contrast, none of the patients reported cardiac symptoms in terms of arrhythmias or heart failure. Laboratory work-up, ECG and echocardiography (including left ventricular function) remained unremarkable at all visits. At the time being, the clinical impact of our observations cannot be definitely assessed. Given the prognostic value of mIBG scintigraphy in the development of clinically significant heart disease, further monitoring of patients with pathological HMR might be necessary to further investigate potential long-term consequences, such as COVID-induced heart failure, especially in the light of the unknown sequelae of the viral infection, better known as long-COVID.

This study has various limitations. First, the analysis included only a relatively small number of patients, thus limiting statistical power. Second, due to the ongoing pandemic at the time point of patient enrollment, no control group without COVID-19 or a viral infection other than COVID-19 (, e.g. influenza or other cardiotropic viruses) was available to assess the impact of this condition on the cardiac sympathetic system. In addition, only correlations to echocardiography and serum measurements were available. To investigate potential consequences of the subclinical condition of increased cardiac sympathetic activity, a correlation of mIBG scintigraphy to cardiac magnetic resonance imaging would have been helpful. Last, the cut-off values used for HMR have been initially established in heart failure patients and lack validation in a non-heart failure population. However, given the fact that we included subjects without pre-existing cardiovascular disease, the current approach might even underestimate pathologic findings in this patient population.

In conclusion, this prospective trial is a first report on cardiac sympathetic dysfunction in COVID-19 patients. Further follow-up and validation in larger, multi-center trials is necessary to investigate potential long-term consequences.

## Data Availability

Data generated or analyzed during the study are available from the corresponding author by request.

## Abbreviations

BMI: Body mass index
HMR: Heart-to-mediastinum ratio
LEHR: Low-energy-high-resolution collimator
LVEF: Left ventricular ejection fraction
mIBG: Metaiodobenzylguanidine
NYHA: New York Heart Association
ROI: Region of interest
